# HIV-1 Tat and Heparan Sulfate Proteoglycans Orchestrate the Setup of *in Cis* and *in Trans* Cell-Surface Interactions Functional to Lymphocyte Trans-Endothelial Migration

**DOI:** 10.3390/molecules26247488

**Published:** 2021-12-10

**Authors:** Chiara Urbinati, Maria Milanesi, Nicola Lauro, Cinzia Bertelli, Guido David, Pasqualina D’Ursi, Marco Rusnati, Paola Chiodelli

**Affiliations:** 1 Experimental Oncology and Immunology, Department of Molecular and Translational Medicine, University of Brescia, 25123 Brescia, Italy; chiara.urbinati@unibs.it (C.U.); m.milanesi006@unibs.it (M.M.); nik_lauro@hotmail.it (N.L.); 2 Department of Cellular, Computational and Integrative Biology, University of Trento, 38123 Trento, Italy; cinzia.bertelli@unitn.it; 3 Department of Human Genetics, University of Leuven, B-3000 Leuven, Belgium; guido.david@kuleuven.be; 4 Institute for Biomedical Technologies, National Research Council (ITB-CNR), 20090 Segrate, Italy; pasqualina.dursi@itb.cnr.it

**Keywords:** HIV-1 Tat, heparan sulfate proteoglycans, lymphocyte extravasation, integrins, signal transduction, endothelial cells, molecular modelling docking and dynamics

## Abstract

HIV-1 transactivating factor Tat is released by infected cells. Extracellular Tat homodimerizes and engages several receptors, including integrins, vascular endothelial growth factor receptor 2 (VEGFR2) and heparan sulfate proteoglycan (HSPG) syndecan-1 expressed on various cells. By means of experimental cell models recapitulating the processes of lymphocyte trans-endothelial migration, here, we demonstrate that upon association with syndecan-1 expressed on lymphocytes, Tat triggers simultaneously the *in cis* activation of lymphocytes themselves and the *in trans* activation of endothelial cells (ECs). This “two-way” activation eventually induces lymphocyte adhesion and spreading onto the substrate and vascular endothelial (VE)-cadherin reorganization at the EC junctions, with consequent endothelial permeabilization, leading to an increased extravasation of Tat-presenting lymphocytes. By means of a panel of biochemical activation assays and specific synthetic inhibitors, we demonstrate that during the above-mentioned processes, syndecan-1, integrins, FAK, src and ERK_1/2_ engagement and activation are needed in the lymphocytes, while VEGFR2, integrin, src and ERK_1/2_ are needed in the endothelium. In conclusion, the Tat/syndecan-1 complex plays a central role in orchestrating the setup of the various *in cis* and *in trans* multimeric complexes at the EC/lymphocyte interface. Thus, by means of computational molecular modelling, docking and dynamics, we also provide a characterization at an atomic level of the binding modes of the Tat/heparin interaction, with heparin herein used as a structural analogue of the heparan sulfate chains of syndecan-1.

## 1. Introduction

Heparan sulfate proteoglycans (HSPGs) are intimately associated with the cell surface and consist of multiple heparin-like glycosaminoglycan (GAG) chains attached to a core protein associated with the cell membrane either via a transmembrane protein domain or via a glycosyl-phosphatidyl-inositol anchor [1]. Syndecans are the most widely represented family of transmembrane HSPGs with a cytoplasmic domain that interacts with the cytoskeleton and transduces a signal within cells upon binding with their extracellular ligands [2]. Syndecan-1 is considered an AIDS-related B-cell lymphoma diagnostic and prognostic marker [3] and represents the dominant HSPG expressed on the surface of multiple myeloma, cells where it regulates cell growth and survival [4].

HSPGs play important roles in HIV-1 infection and dissemination by governing the setup of cell-surface complexes among virions or viral proteins and multiple receptors co-expressed on the same cell (*in cis* interactions) or exposed on adjacent cells (*in trans* interactions). HSPGs bind the transactivating factor Tat, which is released by HIV^+^ leukocytes [5] as a monomer or a homodimer [6,7]. Extracellular Tat released by leukocytes acts in a paracrine manner on different uninfected cell types, including ECs, where it interacts with HSPGs, inducing the *in cis* recruitment and activation of other signaling receptors, such as integrins, VEGFR2 and chemokine receptors [8,9]. This, in turn, causes a dysfunctional state consisting of enhanced cytokine secretion, adhesiveness and permeability [9], which favors leukocyte adhesion, migration and extravasation [10,11]. Tat also acts in an autocrine manner on the same HIV^+^ lymphocytes, where it promptly tethers to their cell-surface HSPGs. In its homodimeric form, leukocyte-associated Tat maintains the capacity to bind another HSPG expressed on the surface of a facing EC, leading to an *in trans* HSPG/Tat-Tat/HSPG quaternary complex that bridges the two cell types, promoting a mechanical lymphocyte adhesion to the endothelium [6]. Additionally, on the surface of lymphocytes, soluble Tat induces the formation of an *in cis* complex between syndecan-1 and integrin α_v_β_3_, which stimulates their chemotactic migration [12]. In conclusion, Tat and HSPGs orchestrate the setup of various *in cis* and *in trans* multimeric complexes involving different receptors, cytoskeleton components and second messengers that lead to the stimulation of both ECs and HIV^+^ lymphocytes, eventually leading to the extravasation of the latter. In turn, this favors HIV dissemination, the creation of reservoirs of infection in different organs [13] and the rise of AIDS-associated pathologies, such as lymphadenopathies, polyclonal B-cell activation, Kaposi sarcoma, and non-Hodgkin lymphoma [6,12].

After adhesion to appropriate substrata, leukocytes undergo a rapid change in shape consisting of their flattening (a process termed “spreading”). This process, which is an absolute prerequisite for diapedesis [14,15], renders spread leukocytes less susceptible to shear force and increases the area of cell contact and the strength of their adhesion. Additionally, during spreading, leukocytes emit “membrane ruffles” with which they “scan” the substrate for counter-receptors and chemokines that will direct their migration [14,15].

In conclusion, besides HSPGs, Tat needs to bind simultaneously to other receptors in order to exert its different biological activities. This is somehow unexpected, due to the small dimension of Tat protein, which is, however, characterized by the presence of distinct functional domains on its surface. These distinct functional domains include: the cysteine-rich region _22_CTNCYCKKCCFHCOVC_37_, involved in Tat homodimerization [16,17] and binding to chemokine receptors CCR2 and CCR3 [18]; the basic region _49_RKKRRQRRR_57_, accredited as the major region responsible for Tat binding to heparin/HSPGs [19] and to VEGFR2 [8,20]; and the _78_RGD_80_ integrin-binding motif [21,22,23].

Here, by exploiting biochemical assays, cell culture models and computational studies, we provide new biological and structural insights into the mechanisms by which HIV-1 Tat and HSPGs act at the interface between endothelium and lymphocytes, orchestrating the engagement of various Tat receptors and inducing acquisition of phenotypes by the two cell types, which are functional to lymphocyte extravasation.

## 2. Results

### 2.1. Experimental Studies

#### 2.1.1. Lymphocyte Adhesion and Spreading onto Substrate-Immobilized Tat

To assess whether lymphocytes undergo spreading after adhesion to substrate-immobilized Tat and to evaluate the involvement of HSPGs in such a process, we exploited the Namalwa cells (NCs) model. These are B-lymphoid cells that, in their parental form or when transfected with an empty vector (EV-NCs), do not express significant levels of HSPGs [24] on their surface and do not adhere to plastic-immobilized Tat or to an EC monolayer enriched with Tat, a capability that is instead acquired when transfected to overexpress syndecan-1 on their surface (SYN-NCs) [6].

**Figure 1:**
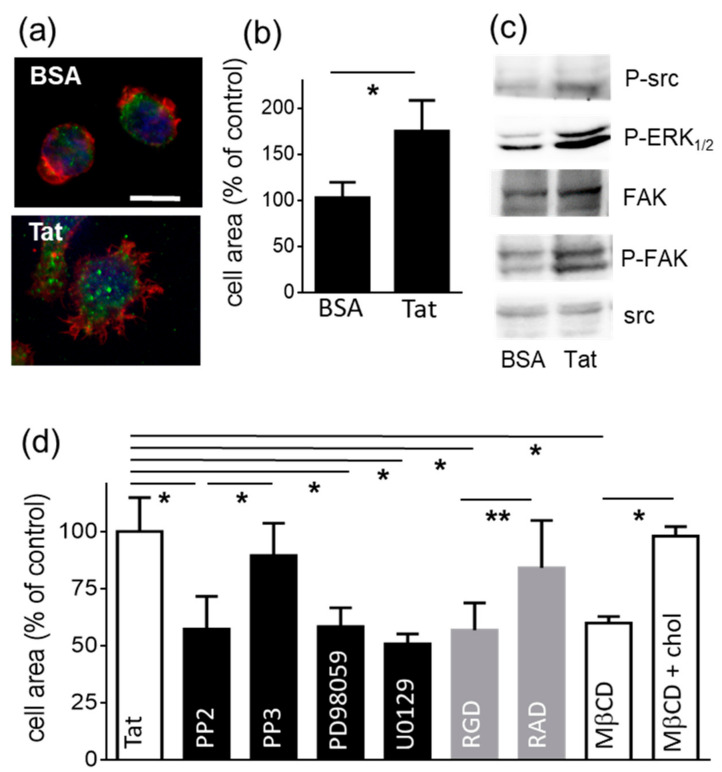
Substrate-immobilized Tat induces SYN-NC spreading. (**a**) Representative microphotographs of SYN-NCs adherent to BSA or to Tat immunostained for actin (red), paxillin (green) and nuclei (blue) (scale bar 5 µm). (**b**) SYN-NC area measured in Tat-adherent cells expressed as mean percentage ± S.E.M. in respect to the area of BSA-adherent cells. * *p* < 0.0001, Student *t*-test. (**c**) Tat-adherent SYN-NCs were analyzed in WB. The intensity of each band was normalized with respect to the corresponding control protein. The statistical significance of Tat-dependent activation was then calculated and reported in Table S1. (**d**) The area of SYN-NCs was measured in 30 to 50 cells, which were allowed to adhere and spread onto Tat in the presence of the indicated inhibitors (chol: cholesterol) and expressed as mean percentage ± S.E.M. in respect to Tat-adherent cells in the absence of any inhibitors. * *p* < 0.01, ** *p* < 0.001, one-way ANOVA.

SYN-NCs were then assayed for their capacity to undergo spreading when adherent to plastic-immobilized Tat. Importantly, SYN-NCs poorly adhere to plastic-immobilized BSA (used as a negative control) in respect to Tat [6]. Additionally, the few BSA-adherent cells retain a rounded shape (Figure 1a). At variance, when seeded onto Tat, they spread (Figure 1a), increasing by more than 1.5 times the substrate contact area in respect to BSA-adherent cells (Figure 1b).

Cell spreading depends on the reorganization of cytoskeleton components, such as actin and paxillin [25]. As shown in Figure 1a, in Tat-adherent and spread SYN-NCs, actin accumulates at the cell periphery, in structures that can be tentatively identified as membrane ruffles. These are actin-rich membrane protrusions found in activated, migrating leukocytes [26,27]. Additionally, in Tat-adherent and spread SYN-NCs, paxillin localizes in discrete, point-like structures attributable to focal adhesion plaques. All these processes depend on syndecan-1 since EV-NCs do not adhere to substrate-immobilized Tat [6].

The engagement of syndecan-1 by soluble Tat induces the activation of src, extracellular signal-regulated kinases 1/2 (ERK_1/2_) and focal adhesion kinase (FAK) [12]. We thus decided to investigate whether substrate-immobilized Tat also retains the capacity to transduce a signal and activate adherent SYN-NCs. As shown in Figure 1c, src, ERK_1/2_ and FAK are activated in SYN-NCs following adhesion to substrate-immobilized Tat.

The involvement of these second messengers in the process of SYN-NCs spreading onto Tat was then evaluated by using a series of specific chemical inhibitors. Importantly, preliminary experiments demonstrated that none of the inhibitors used affects the number of SYN-NCs that adhere to Tat (supplementary material, Figure S1). As shown in Figure 1d, src inhibitor PP2 prevents SYN-NCs from spreading onto Tat, while its negative control, PP3, is ineffective in the same experimental conditions. Additionally, ERK_1/2_ inhibitors PD98059 and U0129 prevent SYN-NCs from spreading onto Tat. Importantly, DMSO (0.1%), which is used as vehicle for PP2, PP3 and PD98059, does not affect SYN-NCs spreading onto Tat (data not shown). FAK activation greatly depends on integrin engagement by extracellular ligands. Furthermore, soluble Tat binds to integrin α_v_β_3_, promoting its *in cis* coupling with syndecan-1 [12]. On these bases, we decided to evaluate whether, besides Syndecan-1, integrin engagement by substrate immobilized Tat is also necessary to induce SYN-NC spreading. As shown in Figure 1d, the pan-integrin inhibitor RGD effectively prevents SYN-NCs from spreading onto Tat, while the negative control, RAD, is ineffective in the same experimental conditions.

Lipid rafts are discrete cell-membrane structures enriched with cholesterol and glycosphingolipids where various signaling receptors, including syndecans [28] and integrins [29], accumulate and cross talk. Lipid rafts are typically found at the leading edge of migrating macrophages and have been implicated in cell adhesion, cytoskeleton organization, spreading, migration and extravasation [29,30]. We then wondered whether lipid rafts were necessary for SYN-NCs to spread onto Tat. For this purpose, SYN-NCs were treated with the lipid-raft-disrupting agent 2,6-di-*O*-methyl-b-ciclodextrin (MβCD) before they were allowed to adhere to immobilized Tat. As shown in Figure 1d, MβCD prevents the spreading of Tat-adherent cells. The specificity of the inhibitory effect is proven by the observation that the spreading of MβCD-treated cells is fully reverted by cholesterol repletion (Figure 1d). Moreover, MβCD does not inhibit the number of SYN-NCs that adhere to substrate-immobilized Tat (supplementary material, Figure S1).

In conclusion, following their syndecan-1-dependent adhesion to substrate-immobilized Tat, lymphocytes undergo cytoskeleton organization, integrin engagement in lipid rafts and the activation of different second messengers, which are all required for their active spreading onto the surface.

**Figure 2:**
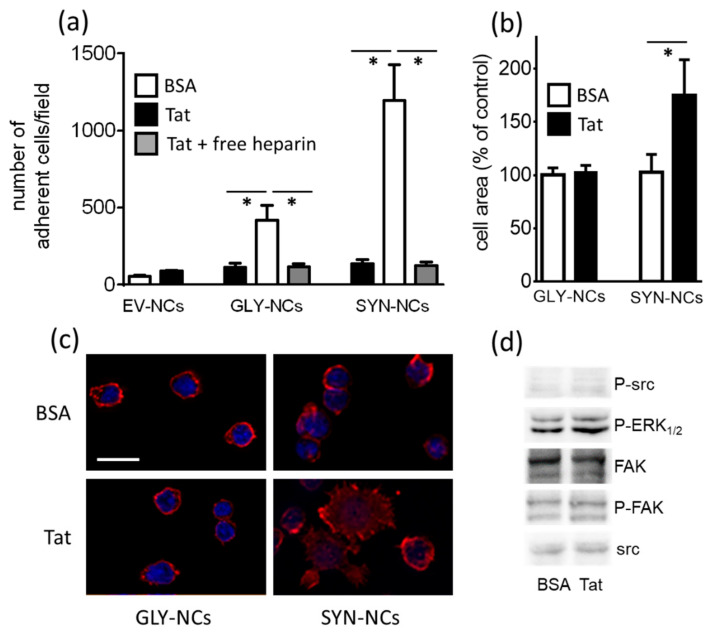
Comparison between GLY-NC and SYN-NC adhesion and spreading onto substrate-immobilized Tat. (**a**) GLY-NCs and SYN-NCs were seeded onto plastic-immobilized BSA or Tat in the absence or presence of free heparin (290 μM). After 2 h, adherent cells were counted. Results are expressed as mean ± S.E.M. of the number of adherent cells/well. * *p* < 0.01, one-way ANOVA. (**b**) The area of Tat-adherent GLY-NCs or SYN-NCs was measured and expressed as mean percentage ± S.E.M. in respect to the area of BSA-adherent cells. * *p* < 0.01, one-way ANOVA. (**c**) Representative microphotographs of GLY-NCs or SYN-NCs adherent to BSA or Tat immunostained for actin (red) and nuclei (blue) (scale bar 10 µm). (**d**) Tat-adherent GLY-NCs were analyzed in WB for phosphorylated (P) src, ERK_1/2_ and FAK. The intensity of each band was normalized with respect to the corresponding control protein. The statistical significance of Tat-dependent activation was then calculated and reported in Table S1.

SYN-NC adhesion to Tat is not affected by inhibitors of integrins, src, ERK_1/2_ or MβCD (Figure S1). At variance, these same inhibitors significantly inhibit the subsequent process of spreading of adherent cells (Figure 1d). These results suggest that the initial adhesion of SYN-NCs to substrate-immobilized Tat is “mechanically” mediated by HSPGs, while the subsequent process of spreading requires the active engagement, activation and redistribution of other membrane receptors and intracellular second messengers. This hypothesis prompted us to compare the process of cell adhesion and spreading mediated by transmembrane syndecan-1 that directly transduces an activation signal within the cell [31] with the GPI-anchored glypican-1 that is instead devoid of transducing capacity. To this end, we exploited glypican-1-overexpressing Namalwa cells (GLY-NCs) and control EV-NCs. Importantly, SYN-NCs and GLY-NCs express similar levels of the two respective HSPGs, while EV-NCs express negligible levels of HSPGs in respect to the two clones mentioned above [24]. As shown in Figure 2a, EV-NCs show a very limited capacity to adhere to Tat. Furthermore, this adhesion is aspecific, being quantitatively similar to that on BSA. On the contrary, the surface expression of glypican-1 or syndecan-1 confer to Namalwa cells the capacity to adhere to Tat (Figure 2a). Finally, free heparin, which acts as HSPG antagonist, inhibits the adhesion of both GLY-NCs and SYN-NCs to Tat. Taken together, these results indicate that heparan sulfate (HS) chains mediate Namalwa cell adhesion to Tat, independently of the core protein with which they are associated. We then evaluated the spreading of Tat-adherent GLY-NCs and SYN-NCs. As shown in Figure 2b,c, only Tat-adherent SYN-NCs, but not GLY-NCs, increase their substrate contact area in respect to BSA-adherent cells (Figure 1b). Finally, at variance with the above observations of SYN-NCs cells, the intracellular second messengers src, ERK_1/2_ and FAK are not activated in Tat-adherent GLY-NCs (Figure 2d).

Taken together, these data indicate that GAG chains of both glypican-1 and syndecan-1 are enough to mediate “mechanical” cell adhesion to Tat, while the more complex process of cell spreading, which requires active signal transduction, can be mediated only by syndecan-1 that possesses a transmembrane and intracellular portion able to activate intracellular second messengers.

#### 2.1.2. Tat-Presenting Lymphocytes Adhere and Spread onto the Endothelium

**Figure 3:**
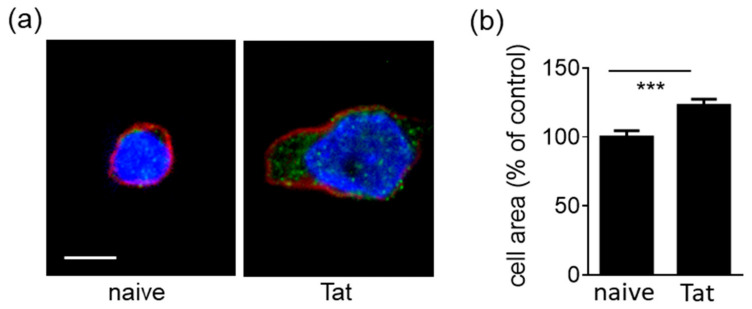
Tat tethered to syndecan-1 of lymphocytes promotes cell spreading onto an EC monolayer. (**a**) Representative microphotographs of naïve or Tat-presenting SYN-NCs adherent to the endothelial monolayer and immunostained with the specific probe for F-actin SiR-actin (red), phosphorylated FAK (green) and nuclei (blue) (scale bar 5 µm). (**b**) The area of adherent Tat-presenting SYN-NCs was measured in 40 to 60 cells and expressed as mean percentage ± S.E.M. in respect to naive cells. *** *p* < 0.001, Student *t*-test.

Once secreted, Tat tends to remain associated to the HSPGs of the surface of the same producing HIV^+^ lymphocytes where, in its homodimeric form, it still induces adhesion to the endothelium by interacting with others HSPGs present on the endothelial surface [6]. To experimentally reproduce this process and assess its consequences with respect to lymphocyte spreading, Tat was allowed to associate to the surface of SYN-NCs, as described in Material and Methods. Dot blot analysis demonstrated that in the adopted experimental conditions, a significant amount of Tat remains effectively associated to SYN-NCs (Figure S2), which is biologically active since it is able to increase by about three times the capacity of the cells to adhere to the endothelium [6]. On this basis, cells that were not exposed to Tat (naïve SYN-NCs) or that were coated with Tat (Tat-presenting SYN-NCs) were seeded onto an EC monolayer and evaluated for their capacity to spread. As shown in Figure 3a, the few naïve SYN-NCs that adhere to the EC monolayer retain a rounded shape, while Tat-presenting SYN-NCs spread, increasing their area of contact with the endothelium in a statistically significant way (Figure 3b). As observed for SYN-NCs adherent to plastic-immobilized Tat, as well as Tat-presenting SYN-NCs adherent to the endothelium, FAK undergoes phosphorylation, localizing in discrete, point-like structures (Figure 3a) resembling those observed for paxillin immunostaining (Figure 1a) and already attributed to focal adhesion plaques. Accordingly, FAK accumulates in these structures in substrate-adherent leukocytes [32].

#### 2.1.3. Tat-Presenting Lymphocytes Activate ECs

**Figure 4:**
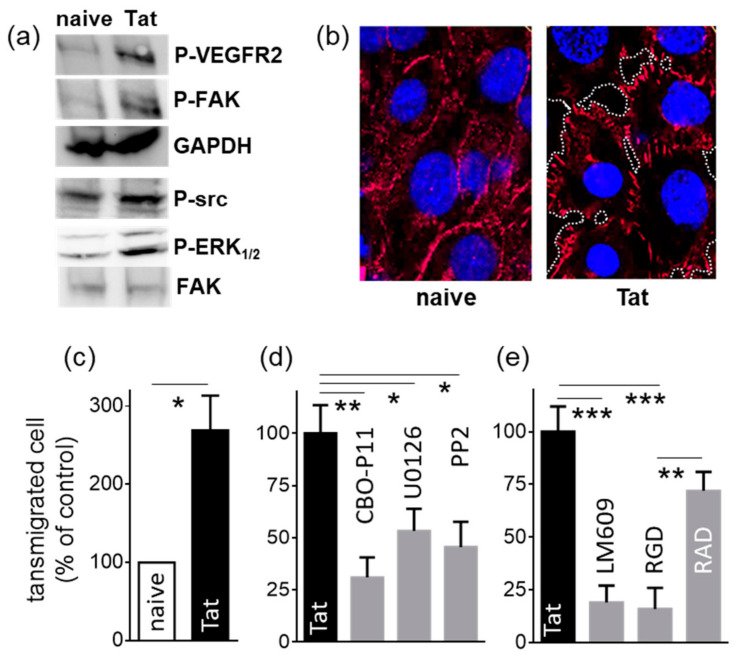
EC activation by Tat-presenting lymphocytes. (**a**) MAEc-VEGFR2 were exposed to naive or Tat-presenting SYN-NCs for 2 h at 37 °C. Then, SYN-NCs were removed by extensive washing, and the EC monolayer was analyzed by WB for the phosphorylation of the indicated proteins. The intensity of each band was normalized with respect to the corresponding control protein. The statistical significance of Tat-dependent activation was then calculated and reported in Table S1. (**b**) Representative microphotographs of EC monolayers exposed to naive or Tat-presenting SYN-NCs, fixed and immunostained for VE-cadherin (red) and nuclei (DAPI, blue). In the right microphotograph, areas uncovered by EC retraction are indicated by white dotted lines. (**c**) Control (naïve) or Tat-presenting SYN-NCs (Tat) were assessed for their capacity to migrate across an EC monolayer in response to CXCL12 (25 nM). Data are expressed as % of migrated cells in respect to control ± S.E.M. * *p* < 0.05, Student *t*-test. (**d**,**e**) Tat-presenting SYN-NCs were evaluated for their capacity to migrate in response to CXCL12 (25 nM) across an EC monolayer that was pretreated with the indicated inhibitors. Data are expressed as % of migrated cells in respect to control EC monolayer without any inhibitor (Tat) ± S.E.M. * *p* < 0.05, ** *p* < 0.01, *** *p* < 0.001, one-way ANOVA.

VEGF-A presented *in trans* by HSPGs retains its capacity to bind and activate VEGFR2 on the surface of facing ECs precursors [33]. On the other hand, free Tat engages and activates VEGFR2 and integrin α_v_β_3_ on ECs, triggering the phosphorylation of src, ERK_1/2_ and FAK [8,34]. On these bases, we decided to evaluate whether Tat presented by syndecan-1 of lymphocytes retains the capability of binding signaling receptors and transducing a signal in ECs. As shown in Figure 4a, VEGFR2, src, ERK_1/2_ and FAK are effectively phosphorylated in ECs exposed to Tat-presenting SYN-NCs but not in ECs exposed to naïve SYN-NCs.

VE-cadherin mediates EC homophylic adhesion and regulates the opening and closing of the endothelial barrier [35], regulating vessel permeability [9]. Additionally, VE-cadherin expression, phosphorylation and distribution are regulated in ECs by the VEGF/VEGFR2 system [36,37,38] and by HIV-Tat itself [37,38]. On these bases, we decided to investigate the distribution of VE-cadherin in an EC monolayer exposed to Tat-presenting SYN-NCs. As shown in Figure 4b, in ECs exposed to naïve SYN-NCs, VE-cadherin molecules are evenly distributed at the interjunctional region of the cells, visualizing an uninterrupted cell layer. At variance, the exposure of the EC monolayer to Tat-presenting SYN-NCs induces the redistribution of VE-cadherin in a disorganized pattern, considered a hallmark of VE-cadherin disengagement, with the consequent disruption of cell-cell contacts and the formation of holes between the cells [39,40].

#### 2.1.4. Tat-Presenting Lymphocytes Migrate across the Endothelium

By maintaining the integrity of EC contacts, VE-cadherin acts as a negative regulator of leukocyte extravasation [41]. Accordingly, during inflammation, VE-cadherin expression, phosphorylation and distribution change dramatically, causing the disruption of the endothelial barrier and leukocyte infiltration. On these bases, we wondered whether Tat associated to syndecan-1 of lymphocytes is able to directly promote the extravasation of the same presenting lymphocytes. As shown in Figure 4c, Tat-presenting SYN-NC extravasation across an EC monolayer is significantly higher than that of naïve cells.

Tat binds and activates VEGFR2, which, in turn, regulates endothelial permeability via VE-cadherin [42]. We thus investigated the role of endothelial VEGFR2 in the extravasation of Tat-presenting SYN-NCs by pretreating an EC with CBO-P11, a cyclic peptide reproducing the VEGF sequence 79–93, which acts as a VEGFR2 antagonist [43]. As shown in Figure 4d, CBO-P11 inhibits SYN-NC extravasation. The same inhibitory effect is obtained by pretreating the EC monolayer with U0126 and PP2, which prevent src and ERK_1/2_ activation, respectively, two second messengers downstream to VEGFR2 (Figure 4d).

Disruption of VE-cadherin-mediated EC contacts is an event that occurs both during inflammation-induced vessel permeabilization [39,40] and endothelial pro-angiogenic activation. Accordingly, Tat can induce both EC inflammatory activation and permeabilization and the acquisition of a pro-angiogenic phenotype [44]. At a molecular level, in the presence of Tat, integrin α_v_β_3_ is necessary to induce the phosphorylation of VEGFR2, src and ERK_1/2_, which leads to an EC pro-angiogenic activation [8]. On these bases, we decided to investigate the involvement of EC integrins in the extravasation of Tat-presenting SYN-NCs. As shown in Figure 4e, pretreatment of an EC monolayer with specific anti-α_v_β_3_ integrin antibody LM609 and pan-integrin inhibitor RGD but not its negative control, RAD, inhibits Tat-presenting SYN-NC extravasation, confirming the requirement of EC integrins for the process of Tat-driven lymphocyte extravasation.

### 2.2. Computational Studies

We finally exploited molecular modelling and docking of the interaction of monomeric and homodimeric Tat with heparin in order to gain further insight into the Tat/HSPG interaction, paying attention to the surface exposure of the various Tat functional domains, including the cysteine-rich region, the basic region and the RGD integrin-binding motif. Due to its structural analogies with HS, heparin has been widely used in computational studies as a surrogate to gain insight into the binding of numerous HSPG/protein complexes [45].

**Figure 5:**
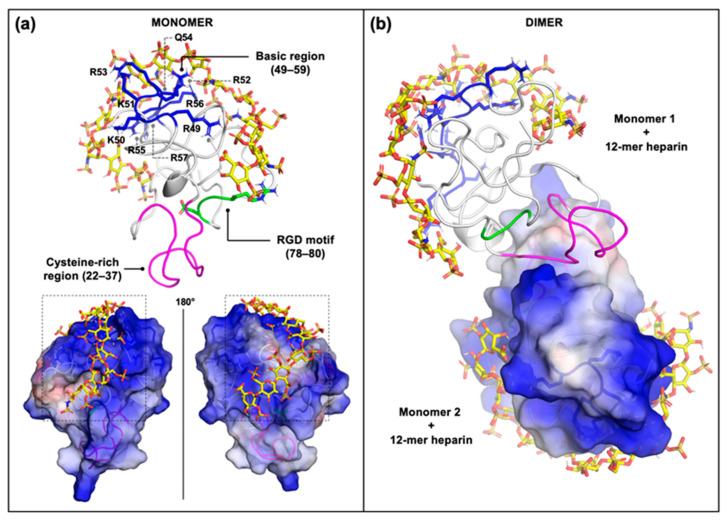
Identification of 12-mer heparin chain (yellow sticks) binding to monomer (**a**) and homodimer (**b**). Tat functional domains are highlighted as follows: heparin binding domain in blue, cysteine-rich domain in magenta and the RGD motif in green. The basic domain residues interacting with heparin are shown in sticks and indicated with the corresponding single letter amino acid code and sequence residue number. The surface of the electrostatic molecular potential of Tat protein is represented with the positively and negatively charged surfaces colored in blue and red, respectively, and the electron-neutral (or non-polar/hydrophobic) surface in white.

Firstly, we prepared the structure of the Tat monomer from HXB2 isolate by a homology modeling strategy, starting from the already characterized structure of the protein from the BRU/LAI strain [46], which shares 93% identity with the amino-acid sequence. The generated model of the Tat monomer shows a folding, similar to the template (Figure 5a), in which the cysteine-rich region is composed of two loops well exposed to the solvent, available for Tat homodimerization [16,17]. Furthermore, the basic domain is characterized by a solvent-exposed extended conformation ready to interact with the known ligand heparin/HSPGs [19] and VEGFR2 [8,20]. Finally, the RGD motif also appears to be located in a solvent-accessible area, available for integrin engagement [21,22,23].

Then, protein-protein docking studies where performed between two monomers to create the Tat homodimer. Consistent with previous experimental results [16,17], our computational studies confirm that Tat homodimerization occurs through the hydrophobic surface of the cysteine-rich regions of the two Tat monomers. In more detail, hydrophobic and aromatic-sulfur interactions occur among residues A_21_, C_22_, Y_26_, C_34_, C_37_, F_38_ and Y_47_ of both monomers. Additionally, polar interactions between Q_35_ and N_24_ of monomers were observed, while residues K_28_ and K_29_ are excluded from the interaction interface (Figure 5b). Importantly, in the Tat homodimer, the basic domains remain exposed on the opposite sites of the surface of the two Tat monomers (Figure 5b), available for heparin/HSPG binding. This is in full agreement with the proposed *in trans* HSPG/Tat-Tat/HSPG quaternary complex that mediate the adhesion of lymphocytes to the endothelium [6].

On this basis, we docked heparin to both the Tat monomer and homodimer. To identify the heparin path, docking simulations used a 4-mer heparin probe and a grid covering the entire monomer or homodimer. Subsequently, the sliding window method was applied to detect the best poses to be concatenated [47], obtaining a final heparin chain of 12-mer bound to both the Tat monomer (Figure 5a) and homodimer (Figure 5b). The interaction of 12-mer heparin with both the Tat monomer and homodimer reveals a network of H-bonds between the SO_3_^−^ and COO^−^ groups of the GAG and the side chains of basic amino acids of the protein (Figure 5). As expected, _49_RKKRRQRRR_57_ residues of the previously characterized main heparin binding domain [19] are involved in this network. In detail, residues R_49_ and the sequence _52_RQRR_56_ interact trough the guanidium group and the amidic group for arginine and glutamine, respectively. Outside the basic domain, the aforementioned chains of residues, Q_17_, K_19_, S_68_, S_70_ S_75_ and R_78_ participate to the hydrogen-bond network between Tat protein and heparin. Finally, with our proposed Tat/heparin model, we were able to demonstrate that, given an appropriate length, the heparin path extends up to the previously described additional binding domain formed by the conformational K_12_K_41_R_78_ triad [48].

## 3. Discussion

During AIDS progression, infected lymphocytes migrate across the endothelial barrier, disseminating HIV-1 infection and triggering the onset of different AIDS-associated pathologies, including kidney [49], brain [50] and cardiovascular [51] diseases and an increased incidence of various tumors [52]. It therefore follows that lymphocyte extravasation can be considered a promising pharmacological target to limit AIDS progression, a goal whose achievement requires knowledge of the mechanisms by which lymphocyte extravasation takes places. The extracellular form of the HIV-1 transactivating factor contributes in many ways to lymphocyte extravasation, firstly by promoting adhesion and then by inducing chemotactic migration. A mandatory passage between lymphocyte adhesion and migration/extravasation is the process of spreading, consisting in lymphocyte flattening, which increases the strength of lymphocyte adhesion and makes them less susceptible to shear force [14,15]. Here, we have demonstrated that, besides its already known capacity to induce lymphocyte adhesion, Tat also mediates the spreading of lymphocytes.

However, these two processes, although closely entangled, are dramatically different. Lymphocyte adhesion to Tat is mediated by both syndecan-1 and glypican-1, being dependent on the direct interaction of Tat with the GAG chains of the two HSPGs. Accordingly, free heparin, a structural analog of HS, acts as an antagonist, inhibiting lymphocyte adhesion to Tat. Additionally, glypican-1 shows a lower adhesive capacity with respect to syndecan-1, despite the similar expression level of the two HSPGs on GLY-NCs and SYN-Ns [24] and their core proteins bearing an equal number (2–3) of GAG-attachment sites [53]. This is likely due to the fact that the HS chains of syndecan-1 are longer that those of glypican-1 [54], likely increasing the Tat-interactive capacity of the former in respect to the latter. Moreover, in syndecan-1, HS chains are terminally attached to the ectodomain, suitably available for ligand binding. At variance, in glypican-1, HS chains are attached near the plasma membrane, with the ectodomain assuming a globular, bulky conformation, which possibly hampers the accessibility of HS chains below [53].

The process of lymphocyte spreading onto Tat can be instead mediated only by syndecan-1 but not by glypican-1. Accordingly, it depends on the activation of different second messengers, which occurs only in lymphocytes that overexpress syndecan-1 but not glypican-1, indicating that the signal transduction mediated by the intracellular tail of syndecan-1 core protein [12] is required for the spreading process. Besides syndecan-1, integrin engagement and activation by Tat are also required for a full lymphocyte spreading, suggesting that although any HSPG is enough to mediate an initial, mechanical lymphocyte adhesion to substrate-immobilized Tat, for a complete cell spreading, integrins are also required, along with a complete signal-transduction pathway generated by its coupling with syndecan-1 [12].

Adhesion, spreading and migration are mandatory but not sufficient for lymphocyte extravasation, which also requires an increase in vessel permeability. In physiological conditions, the integrity of the endothelial barrier is maintained by the concerted action of cytoskeleton components [55] and cell-adhesion molecules, among which VE-cadherin [42] maintains ECs closely associated to each other, forming a continuous monolayer. Accordingly, vascular permeability is increased by the disruption of VE-cadherin organization following appropriate activation of receptors and second signaling, such as VEGFR2, integrins and src [36,56,57] or lipid raft-dependent MMP-2 and MMP-3 activation [58]. It is relevant to note that here, we observed that these cellular structures are indeed involved in VE-cadherin re-organization in lymphocytes adherent to Tat.

So as to favor their dissemination in the host, viruses have evolved the capacity to increase endothelial permeability. This can occur by an abnormal host inflammatory response with a consequent “cytokine storm”, which can even lead to vascular damage [59]. Alternatively, virus proteins released by infected cells can directly induce vascular leak by different mechanisms [60,61]. In particular, HIV-1 Tat has been demonstrated to alter the physiological organization of both the endothelial cytoskeleton [55] and VE-cadherin [37,38]. Here, we demonstrated that Tat-presenting lymphocytes induce the loss of the integrity of the EC monolayer. Accordingly, lymphocyte extravasation depends on the activation of endothelial VEGFR2 and α_v_β_3_, with the consequent transduction of a signal that includes the phosphorylation of src, FAK and ERK_1/2_.

**Figure 6:**
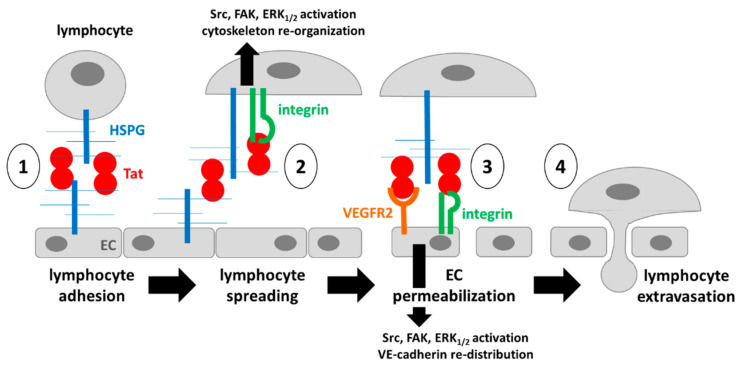
Schematic representation of the setting up of multimeric complexes orchestrated by Tat and HSPGs at the lymphocyte-endothelium interface: (1) *in trans* HSPG/Tat-Tat-HSPG quaternary complex necessary for the preliminary adhesion of lymphocytes to the endothelium; (2) *in cis* HSPG/Tat/integrin on the lymphocyte responsible for cell spreading; (3) *in trans* HSPG-Tat-VEGFR2 and HSPG-Tat-integrin interactions responsible for VE-cadherin redistribution and endothelial permeabilization that, together with the interaction mentioned at point (2), is responsible for lymphocyte extravasation (4).

In conclusion, at the lymphocyte/endothelium interface, Tat association to HSPGs orchestrates the setting up of various *in cis* and *in trans* multimeric complexes among different receptors that trigger a complex signal transduction inside the two cells and, hence, endothelial permeabilization and lymphocyte spreading and extravasation (Figure 6).

Here, molecular modeling and docking were used to characterize the structures of monomeric or homodimeric Tat. Similar studies have been conducted so far only concerning the monomeric form of Tat [48,62,63,64,65], with this study thus representing a significant improvement, which allows the fine characterization of the binding mode of the two cysteine domains during Tat homodimerization. Furthermore, we demonstrated how in the Tat homodimer, the basic domain, _49_RKKRRQRRR_57_, which acts as binding domain for heparin/HSPGs and VEGFR2, remains well exposed on the opposite surface of the two monomers, available for some of the *in cis* or *in trans* multimeric complex schematized in Figure 6. In agreement with these predictions, the _49_RKKRRQRRR_57_ basic domain has been demonstrated to be the major domain responsible for Tat binding to heparin/HSPGs [19]. Accordingly, a mutated form of Tat in which this heparin-binding domain has been “neutralized” by substituting its basic residues with alanine residues does not induce SYN-NC adhesion to the substrate [6].

Subsequent docking studies were then performed between the Tat homodimer and heparin, used here as a structural analogue of the GAG chains of HSPGs [45]. By means of the sliding window method [47], we succeeded in obtaining a model of two separate 12-mer heparin chains that bind the two monomers of the Tat homodimer, in agreement with the *in trans* HSPG/Tat-Tat/HSPG quaternary complex [6]. However, it is also apparent that the two 12-mer heparin chains bound to the Tat homodimers point to one another, suggesting that in vivo, heparin (or HS) chains of suitable length can connect two Tat monomers simultaneously, favoring protein oligomerization, as experimentally demonstrated in previous works [66].

Previous docking simulations of the Tat/heparin complex performed by using the shorter 8-mer heparin allowed for the identification of an alternative conformational heparin-binding domain composed of K_12_, K_41_ and R_78_ [48]. Here, by using the longer 12-mer heparin, it was possible to show that the heparin chain fits in a basic path that can effectively connect the two heparin-binding domains.

The RGD motif mediates the binding of Tat to integrins involved in some of the *in cis* and *in trans* multimeric complexes schematized in Figure 6. Notably, the RGD motif remains exposed on the surface of homodimeric Tat, as already demonstrated in the monomeric form [21,67]. It is finally necessary to understand the availability of the RGD motif in the Tat/heparin complex. Relevantly, the heparin-binding K_12_K_41_R_78_ triad partially overlaps the _78_RGD_80_ motif, and accordingly, Ai and colleagues described how the interaction of heparin/HSPGs with K_12_K_41_R_78_ effectively modulates Tat-driven integrin activation and subsequent cell adhesion [48]. To definitely outline the interplay between Tat, HSPGs and integrins, it will be necessary to perform molecular docking and dynamic simulations of the resulting trimeric complex.

## 4. Materials and Methods

**Reagents:** 86 amino acid HIV-1 Tat was expressed and purified by *Escherichia coli* as glutathione-S-transferase (GST-Tat) [19] fusion protein. GST moiety does not interfere with the heparin-binding, transactivating and cell-adhesive capacity of Tat [68]. Anti-P-FAK Tyr397, anti-FAK, anti P-src Tyr 416 antibodies, CellTracker™ Red CMTPX, tetramethylrhodamine (TRITC)-phalloidin, 4’,6-diamidin-2-fenilindolo (DAPI), Alexa Fluor 488 conjugated to anti-mouse IgG, anti-Alexa Fluor 594 or Alexa Fluor 647 conjugated to goat IgG were obtained from Invitrogen Thermo Fisher Scientific, Waltham, MA, USA. Cyclo(-Arg-Gly-Asp-D-Phe-Val) (cRGDfV) and cyclo(-Arg-Ala-Asp-D-Phe-Val) (cRADfV) peptides were obtained from Bachem, Beidendorf, Switzerland. PP2 (4-amino-5-(4-chlorophenyl)-7-(t-butyl) pyrazolo [3,4-d] pyrimidine), PP3, U0126, VEGF inhibitor CBO-P11, anti-p-src Tyr416 antibody, and anti-αvβ3 antibody LM609 were obtained from Calbiochem, Merck, Darmstadt, Germany. Anti-VE-cadherin and anti-paxillin antibodies were obtained from Santa Cruz Biotechnology, Santa Cruz, CA, USA. Anti P-VEGFR2 Tyr 1175 and anti P-ERK_1/2_ antibodies were obtained from Cell Signaling Technology, Danvers, MA, USA. MβCD and cholesterol were obtained from Merck, Darmstadt, Germany. The fluorogenic SiR-Actin was obtained from Spirochrome AG, Stein am Rhein, Switzerland.

**Cell cultures:** Burkitt lymphoma-derived Namalwa cell clones transfected with an empty vector EV-NCs or with vectors containing Syndecan-1 or Glypican-1 cDNA (SYN-NCs and GLY-NCs, respectively) were cultured in RPMI containing 10% fetal calf serum (FCS), 100 U/mL penicillin, 50 mg/mL streptomycin and 1 mM L-glutamine (Invitrogen, Thermo Fisher Scientific). Transformed fetal bovine aortic endothelial GM7373 cells, from the Human Genetic Mutant Cell Repository, Institute for Medical Research, Camden, NJ, USA, were grown in Dulbecco’s modified minimum essential medium (DMEM) containing 10% FCS. GM7373 cells were transfected to generate stable GM7373-VEGFR2 transfectant cells, as described in [69]. Murine aortic ECs (MAEc) transfected with mouse VEGFR2 were grown in DMEM 10% FCS.

**Namalwa adhesion and spreading assay to immobilized proteins:** Proteins were immobilized to non-tissue culture plastic, as described in [70]. Briefly, aliquots of 100 mM NaHCO3, pH 9.6 (carbonate buffer), containing the adhesive molecule under test were added to polystyrene non-tissue culture plates. After 16 h of incubation at 4 °C, the solution was removed, and wells were washed three times with cold PBS. SYN-NCs, GLY-NCs or EV-NCs (100,000 cells/200 μL of RPMI 1% FCS) were seeded onto the wells containing the coated proteins and allowed to adhere and spread for 2 h at 37 °C in the absence or in the presence of PP2 and PP3 (10 μM), PD98059 (10 μM), U0129 (5 μM), cRADfV and cRGDfV (10 μM). MβCD (25 μM), cholesterol (chol) (400 μg/mL). Then, wells were washed three times with 2 mM EDTA in PBS, stained with crystal violet and photographed under an inverted microscope (Olympus IX51 connected to an Olympus Camedia C4040Z digital camera, Olympus Optical Co., LTD, Tokyo, Japan). The images were digitalized. For each photograph, 6 microscopic fields (×400 magnification) were chosen randomly of which the number of adherent cells was counted or the area of each adherent cell was evaluated by ImageJ software (National Institute of Health, Bethesda, MD, USA).

**SYN-NCs loading with Tat:** To experimentally reproduce the HIV^+^ positive lymphocytes that present HSPG-associated Tat on their surface, aliquots of SYN-NCs cells (1 × 10^6^ cells/500 μL of RPMI 0% FCS) were incubated for 30 min at 37 °C with DMEM containing GST-Tat (550 nM) and then extensively washed with PBS to remove unbound Tat. The effective association of Tat to HSPGs of the lymphocyte surface was assessed by dot blot analysis, as described in Figure S2.

**SYN-NC adhesion and spreading assay to EC monolayers:** Monolayers of GM7373 and GM7373-VEGFR2 were starved for 6 h in DMEM. Naïve or Tat-presenting SYN-NCs were incubated for 30 min with 1 μM CellTracker™ Red CMTPX dye. SYN-NCs were allowed to adhere onto the EC monolayers for 2–4 h at 37 °C for phosphorylation assay and adhesion assay, respectively. Then, wells were washed, and adherent cells were fixed and photographed under a Zeiss Axiovert 200 M epifluorescence microscope equipped with Apotome and a Plan-Apochromat ×63/1.4 NA oil objective (Carl Zeiss, Gottingen, Germany). For each experimental condition, the area of each adherent cell was measured in 40 to 60 cells by ImageJ software.

**Immunofluorescence assay:** Namalwa cells adherent to plastic-immobilized BSA or Tat were fixed, permeabilized, saturated with PBS containing 3% BSA and stained with 5 U/mL TRITC-phalloidin to decorate actin and incubated for 1 h with anti-paxillin antibody (1:100), followed by a 45 min incubation with Alexa Fluor 488-conjugated anti-mouse IgG to decorate paxillin. Nuclei were counterstained with DAPI. In other experiments, MAEc-VEGFR2 (60,000/cm^2^) were seeded onto IBIDI 8-well micro-slides (IBIDI, GmbH, Gräfelfing, Germany) and allowed to form a monolayer. SYN-NCs were incubated for 30 min at 37 °C with DMEM containing 550 nM GST-Tat and 1 μM SiR-Actin. SYN-NCs were allowed to adhere to ECs for 4 h at 37 °C. Then, wells were washed, as described, and cells were fixed, permeabilized, saturated with PBS containing 3% BSA and stained overnight with anti-VE-Cadherin antibody (1:350), followed by anti-goat IgG conjugated with Alexa Fluor 647 and anti-p-FAK antibody (1:75), followed by Alexa Fluor 488-conjugated anti-rabbit IgG. Nuclei were counterstained with DAPI. Cells were photographed under a Zeiss Axiovert 200 M epifluorescence microscope equipped with Apotome and a Plan-Apochromat ×63/1.4 NA oil objective (Carl Zeiss, Gottingen, Germany).

**VEGFR2 or second messenger phosphorylation assays:** SYN-NCs adherent to immobilized Tat or EC monolayers that had been exposed to Tat-presenting SYN-NCs were lysed in lysis buffer (TRIS-HCl pH 7 50 mM, NaCl 150 mM, Triton X-100 1%, BriJ 0.1%). Then, 100 μg protein/sample was resuspended in reducing SDS-PAGE sample buffer, boiled for 5 min at 95 °C, separated by SDS-10% PAGE and analyzed by Western blot for the phosphorylated form of VEGFR2, ERK_1/2_, FAK or src. Uniform loading was confirmed with anti-FAK, anti-src or anti-GAPDH antibody. The intensity of each band was normalized with respect to the corresponding control protein. The statistical significance of Tat-dependent activation was then calculated and reported in Table S1.

**SYN-NCs transmigration assay:** GM7373-VEGFR2 (40,000 cells) were seeded on collagen-coated 24-well Transwell culture inserts (6.5 mm diameter clear polycarbonate membrane, 5 μm pores; Costar, Corning, NY, USA) and cultured in complete medium to form a complete monolayer. SYN-NCs were incubated for 30 min at 37 °C with DMEM with or without 550 nM Tat; SYN-NCs were washed with PBS to remove unbound Tat; SYN-NCs (500,000 cells/200 μL of DMEM 0.4% FCS) were then placed in the upper chamber of each Transwell insert. Assay medium (500 μL) containing CXCL12 (25 nM) (Peprotech, Rocky Hill, NJ, USA) was added in the lower chamber. After 4 h at 37 °C, migrated cells were collected by centrifugation and counted in a Burker chamber. In some experiments, the ECs monolayer was pretreated for 30 min at 37 °C with cRGDfV or cRADfV (30 µM), anti- αvβ3 antibody LM609 (25 µg/mL), VEGF inhibitor CBO-P11 (10 µM), U0126 or PP2 (10 µM).

**Statistics:** Statistical analyses were performed using the Prism 6 statistical package. Student’s *t*-test or one-way analysis of variance was followed by Bonferroni multiple comparison post-test. Data were expressed as mean ± SEM. Differences were considered significant when *p* < 0.05.

**Computational studies:** Tat structure (isolate BRU/Lai, UniProt P04610) was used as template to create a 3D model of Tat isolate HXB2 /UniProt P04608) using the MODELLER 9.17 program [71]. Ten structures were generated by the program, and the best structure was selected based on the lowest DOPE assessment score. To model the Tat homodimer, the automatic protein-protein docking ClusPro web server with the multimeric option was used [72]. The resulting complexes belonging to the most represented cluster were analyzed by visual inspection and chosen.

*Heparin path identification:* Blind docking simulations were performed Autodock Vina [73] with a grid size of 40 × 40 × 40 for the monomer and 70 × 50 × 70 for the homodimer and exhaustiveness set at 24. 4-mer heparin modeled based on its NMR available solution structure (PDB entry 1HPN) was used as ligand to identify heparin-binding regions on Tat, which were then filtered by best score and visual inspection to achieve a traced heparin path.

*Incremental docking and heparin modelling:* The 4-mer heparin probe was subjected to local docking simulation by Autodock Vina along the traced heparin path in Tat. The “sliding window method” was set up to create a sequence of overlapping sliding grids. Local docking poses were filtered for free energy of binding, cluster size and correct orientation. The aligned 4-mer heparin probes were joined by 1 → 4 glycosidic linkages using Pymol [http://www.pymol.org/pymol (accessed on 1 June 2021)]. Gasteiger-Hückel charges were assigned to the sugar and then minimized by Chimera [74], obtaining heparin chains of increasing length.

## Data Availability

Not applicable.

## Supplementary Materials

The following supplementary materials are available online, Table S1: Quantification of the Tat-dependent activation of the various receptors and second messengers in the different experimental conditions adopted. Figure S1: Inhibitors of integrin, src, ERK_1/2_ and lipid raft formation do not affect SYN-NCs adhesion to Tat. Figure S2: Coating of the lymphocyte surface with Tat.
