# Supplementary material for: HIV-1 Tat and Heparan Sulfate Proteoglycans Orchestrate the Setup of in Cis and in Trans Cell-Surface Interactions Functional to Lymphocyte Trans-Endothelial Migration
Source: Molecules. 2021 Dec 10;26(24):7488. doi: 10.3390/molecules26247488 (PMC8705413; doi:10.3390/molecules26247488)
Supplement: Supplementary file 1 [file molecules-26-07488-s001.zip › molecules-1478427-supplementary material.pdf]

Supplementary materials

HIV-1 Tat and Heparan Sulfate Proteoglycans Orchestrate the Setup of *in Cis* and *in Trans* Cell-Surface Interactions Functional to Lymphocyte Trans-Endothelial Migration

Chiara Urbinati [1], Maria Milanesi [1], Nicola Lauro [1], Cinzia Bertelli [2], Guido David [3], Pasqualina D'Ursi [4], Marco Rusnati [1,*] and Paola Chiodelli [1,*]

[1] Experimental Oncology and Immunology, Department of Molecular and Translational Medicine, University of Brescia, 25123 Brescia, Italy; chiara.urbinati@unibs.it (C.U.); m.milanesi006@unibs.it (M.M.); nik_lauro@hotmail.it (N.L.)
[2] Department of Cellular, Computational and Integrative Biology, University of Trento, 38123, Povo, Trento, Italy; cinzia.bertelli@unitn.it
[3] Department of Human Genetics, University of Leuven, B-3000 Leuven, Belgium; guido.david@kuleuven.be
[4] Institute for Biomedical Technologies, National Research Council (ITB-CNR), 20090, Segrate (MI), Italy; pasqualina.dursi@itb.cnr.it
* Correspondence: marco.rusnati@unibs.it (M.R.); paola.chiodelli@unibs.it (P.C.)

Table S1. Quantification of the Tat-dependent activation of the various receptors and second messengers in Table 1. c and 2d) or Tat versus naïve (Figure 4a). p-value: * <0.05, ** < 0.01. n.d., not determined.

Receptors/Second Messengers	Figure 1c	Figure 2d	Figure 4a
**P-ERK1/2**	174 ± 14 **	108 ± 8	347 ± 36 **
**P-src**	150 ± 28	109 ± 9	132 ± 10 *
**P-FAK**	192 ± 32 *	87 ± 10	165 ± 36
**P-VEGFR2**	n.d.	n.d.	240 ± 43 *

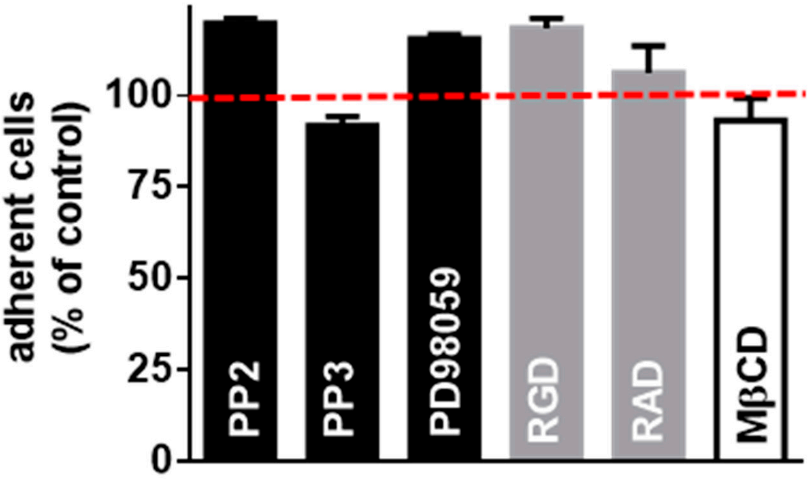

Figure S1. Inhibitors of integrin, src, ERK1/2 and lipid raft formation do not affect SYN-NCs adhesion to Tat. SYN-NCs were seeded onto plastic-immobilized Tat in the absence or in presence of the indicated compounds (same doses used for cell spreading assays). After 2 hours, adherent cells were counted and expressed as mean percentage ± S.E.M. in respect to the number of cells that adhered to Tat in the absence of any inhibitor (red dotter line).

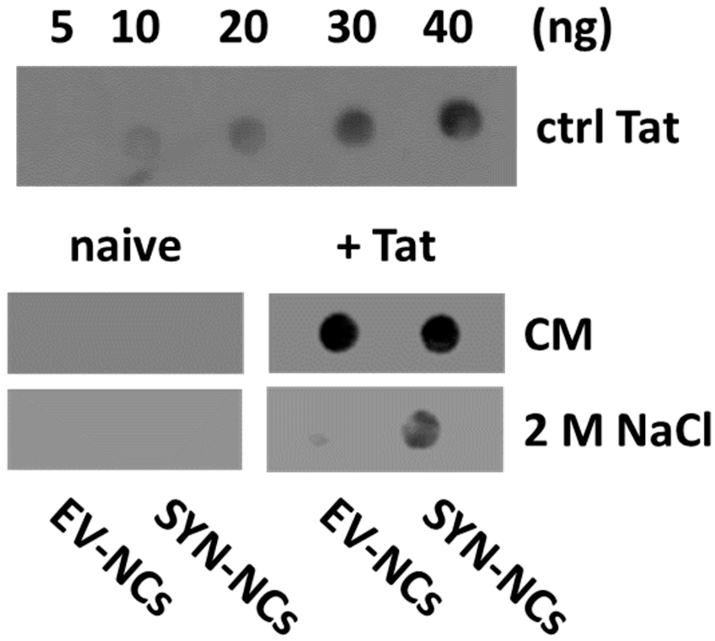

Figure S2. Coating of the lymphocyte surface with Tat. Samples (1x10[6] cells) of EV-NCs or SYN-NCs were incubated for 30 min at 37° C in the absence or in the presence of Tat (550 nM). At the end of incubation, conditioned medium was removed and cells extensively with PBS to remove unbound Tat. Then, cells were washed twice with 1.5 M NaCl., a treatment that is known to detach Tat form HSPGs binding [1]. The entire volume of the PBS washing, the 1,5 M NaCl washing and conditioned media (CM) from the different experimental conditions were subjected to dot blot analysis with anti-Tat antibodies.

References

1. Urbinati, C.; Bugatti, A.; Oreste, P.; Zoppetti, G.; Waltenberger, J.; Mitola, S.; Ribatti, D.; Presta, M.; Rusnati, M. Chemically sulfated Escherichia coli K5 polysaccharide derivatives as extracellular HIV-1 Tat protein antagonists. FEBS letters 2004, 568, 171-177, doi:10.1016/j.febslet.2004.05.033.
